# The PHAR-QA Project: Competency Framework for Pharmacy Practice—First Steps, the Results of the European Network Delphi Round 1

**DOI:** 10.3390/pharmacy3040307

**Published:** 2015-11-17

**Authors:** Jeffrey Atkinson, Kristien De Paepe, Antonio Sánchez Pozo, Dimitrios Rekkas, Daisy Volmer, Jouni Hirvonen, Borut Bozic, Agnieska Skowron, Constantin Mircioiu, Annie Marcincal, Andries Koster, Keith Wilson, Chris van Schravendijk

**Affiliations:** 1 Pharmacology Department Lorraine University, Pharmacolor Consultants Nancy, 12 rue de Versigny, Villers 54600, France; 2 Department of Pharmaceutical and Pharmacological Sciences, Research group of In Vitro Toxicology and Dermato-Cosmetology, Vrije Universiteit Brussel, Laarbeeklaan 103, Brussels 1090, Belgium; E-Mail: kdepaepe@vub.ac.be; 3 Faculty of Pharmacy, University of Granada (UGR), Campus Universitario de la Cartuja s/n, Granada 18701, Spain; E-Mail: sanchezpster@gmail.com; 4 School of Pharmacy, National and Kapodistrian University Athens, Panepistimiou 30, Athens 10679, Greece; E-Mail: rekkas@pharm.uoa.gr; 5 Pharmacy Faculty, University of Tartu, Nooruse 1, Tartu 50411, Estonia; E-Mail: daisy.volmer@ut.ee; 6 Pharmacy Faculty, University of Helsinki, Yliopistonkatu 4, P.O. Box 33-4, Helsinki 00014, Finland; E-Mail: jouni.hirvonen@helsinki.fi; 7 Faculty of Pharmacy, University of Ljubljana, Askerceva cesta 7, Ljubljana 1000, Slovenia; E-Mail: Borut.Bozic@ffa.uni-lj.si; 8 Pharmacy Faculty, Jagiellonian University, UL, Golebia 24, Krakow 31-007, Poland; E-Mail: askowron@cm-uj.krakow.pl; 9 Pharmacy Faculty, University of Medicine and Pharmacy “Carol Davila” Bucharest, Dionisie Lupu 37, Bucharest 020021, Romania; E-Mail: constantin.mircioiu@yahoo.com; 10 European Association of Faculties of Pharmacy, Faculty of Pharmacy, Université de Lille 2, Lille 59000, France; E-Mail: annie.marcincal@univ-lille2.fr; 11 European Association of Faculties of Pharmacy, Department Pharmaceutical Sciences, Utrecht University, P.O. Box 80082, 3508 TB Utrecht, The Netherlands; E-Mail: A.S.Koster@uu.nl; 12 School of Life and Health Sciences, Aston University, Birmingham B4 7ET, UK; E-Mail: k.a.wilson@aston.ac.uk; 13 MEDINE2, Vrije Universiteit Brussel, Laarbeeklaan 103, 1090 Brussels, Belgium; E-Mail: chrisvs@vub.ac.be

**Keywords:** pharmacy, competence, education, practice

## Abstract

PHAR-QA, funded by the European Commission, is producing a framework of competences for pharmacy practice. The framework is in line with the EU directive on sectoral professions and takes into account the diversity of the pharmacy profession and the on-going changes in healthcare systems (with an increasingly important role for pharmacists), and in the pharmaceutical industry. PHAR-QA is asking academia, students and practicing pharmacists to rank competences required for practice. The results show that competences in the areas of “drug interactions”, “need for drug treatment” and “provision of information and service” were ranked highest whereas those in the areas of “ability to design and conduct research” and “development and production of medicines” were ranked lower. For the latter two categories, industrial pharmacists ranked them higher than did the other five groups.

## 1. Introduction

Competences, and resulting learning outcomes, are more meaningful indicators than course content or duration. Furthermore, a profession such as pharmacy is defined by competences that are regularly refined in order to fulfill society’s demands.

The PHAR-QA project [1] will produce a consensual, harmonized competence framework for pharmacy practice to be used as a base for a QA system for evaluation of university pharmacy education and training at the institutional, national and/or European levels. The framework is in line with the European Union (EU) directive 2013/55/EU on sectoral professions [2] and takes into account the diversity of the pharmacy profession as well as the on-going changes in healthcare systems (with an increasingly important role for pharmacists), and in the pharmaceutical industry. The varying impact of these different factors on pharmacy education in the European setting has been described in detail elsewhere [3,4,5].

The PHAR-QA consortium is working essentially within the context of 2 of the 5 pillars of the “pillars and foundations of quality” model of the International Pharmaceutical Federation (FIP) [6] namely “context” and “process”. Regarding context, the internal environment *i.e.*, the department and university levels, is similar in Europe to that of departments in other regions like the USA, Canada or Australia. The external environment *i.e*., the political and legal context is somewhat different. Whilst the EU directive 2013/55/EU aims at ensuring competence for pharmacy practice and gives some indications of how education can be organized to provide such competences, it also, importantly, fixes the minimum requirements for pharmacists wishing to work in a different member state country from that in which they received their education and training. This ensures the fundamental principle of the EU that is free movement across borders. EU directives are governed by comitology the process by which a directive acceptable to all members is produced. Within this context, competence frameworks are needed as a tool for international recognition when dealing with the different educational systems and programs in different EU member states. In healthcare more generally, frameworks are designed as educational and developmental tools used both in academia and in practice, both foundation formation and continuous professional development [7,8,9,10]. This is the case for competence frameworks that are being developed in individual European countries like Serbia [11], Lithuania [12], Ireland [13], and the UK [14].

The second aspect concerns the pillar “process” which in the FIP document cited above includes nine different activities from strategic planning to appraisal and development of academic staff. This article deals specifically with the seventh of these “process” activities: curricular development and improvement. The framework is intended for a European 5-year pharmacy degree.

Under the auspices of EAFP [15], PHAR-QA brought together several of the major players in pharmacy education from “old” and “new Europe”, and from eastern, western, southern and northern Europe (the authors).

The methodology was based similar on that of MEDINE (Medical Education in Europe) [16] in which a framework for medical competences was proposed. Furthermore, PHAR-QA has a representative from MEDINE to help solve the many difficulties of this complex type of project.

In UK English “competence” is defined by the Oxford English Dictionary in four main ways, way 4a being “sufficiency of qualification; capacity to deal adequately with a subject” [17]. In American English, Merriam-Webster’s dictionary defines “competence” as “the ability to do something well” [18]. We have used the word in this way with the additional subdivision of propositions for competences into (1) “knowledge” = “being aware of” and thus capable of applying; and (2) “ability” = “capable of doing”. Thus, our definition of competence is in line with that of the American Council on Credentialing in Pharmacy [19]: “competence is the ability of a pharmacist based on his knowledge and experience to make the right decision in favor of his patient”.

Stakeholders are the major EU pharmacy agencies and associations: PGEU [20], EPSA [21], EAHP [22], and EIPG [23]. PHAR-QA has made contact with pharmacy education QA agencies in the USA (ACPE [24]) and in Australia and New Zealand (PhLOS [25]). This has led to interesting and useful verbal exchange the essence of which has been transcribed into this paper.

## 2. Methodology

**Figure 1:**
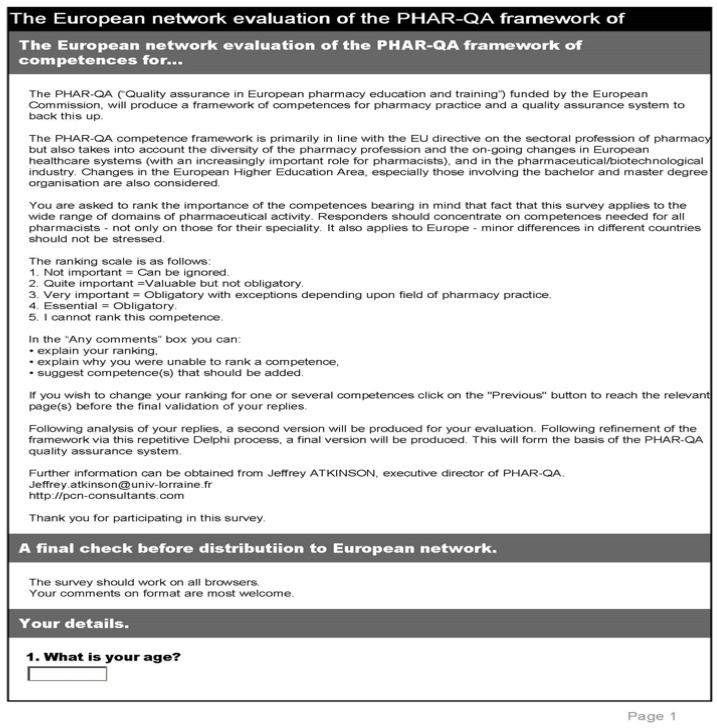
The introductory page of the *surveymonkey* questionnaire.

The two main phases of the PHAR-QA project were (1) 3 Delphi rounds within the consortium (authors of this paper), finishing with the development of the PHAR-QA competence framework; and (2) a European-wide survey to refine the framework in a further 2 Delphi rounds and obtain harmonized EU backing for the framework. Thus, the project uses a modified Delphi approach [26]:

(1) Initial questionnaire—round 1 questionnaire was produced by A. Sanchez-Pozo and D. Rekkas using various references [2,27,28,29,30,31,32,33] together with comments from the other authors.
(2) Evaluation by the consortial expert panel (the authors)—the round 1 questionnaire was modified in three Delphi rounds, the panel providing rankings and comments on what was unclear, missing, or in duplicate, *etc.*, so producing the fourth version. Nine out of thirteen of the panel (authors) are practicing pharmacists in addition to being academics. Several have more than 20 years of experience as practicing pharmacists. Twelve out of thirteen have a long experience of university teaching of pharmacy, in most cases of 25 years or more. One is an expert in medical education. Once terminology issues were resolved there was widespread consensus on the different visions of pharmacy practice.
(3) The fourth version of the questionnaire consisting of 68 propositions for competences for pharmacy practice in 13 clusters was submitted to a large expert panel (academics, students, and pharmacists from all areas of the profession (*n* = 1245).
(4) The analysis of ranking data and comments on the fourth version, gathered using a *surveymonkey* questionnaire [34], will lead to the production of the fifth version. The ranking data and comments on the fourth version are presented in this article. The *surveymonkey* questionnaire (Figure 1) was available online from 14 February 2014 through 1 November 2015 *i.e.*, 8.5 months. Such a long period was required in order to achieve (a modicum of) balance in the distribution of respondents (by occupation, country, age…).
(5) A future second evaluation by the large European wide expert panel will lead to the production of the final QA framework.

It should be noted that the first phase of this Delphi process consists in the production of a concerted, harmonious, position paper by a group of experts; this is the essence of the Delphi process [35]. The second phase—the European wide survey—is aimed at producing a harmonized European position on general competence framework.

There were six questions on the profile of the respondent:

(1) Age
(2) Country of residence
(3) Current occupation: community, hospital or industrial pharmacist, pharmacist working on other area, student, academic
(4) If you are a student, what is your year of enrolment?
(5) If you are a professional (licensed practitioner, academic staff...), how long have you been practicing?
(6) Job title

These were followed by 13 clusters in two major domains with a total in all of 68 competences (see Appendix). Questions in clusters 7 through 11 were concerned with personal competences and in clusters 12 through 19 with patient care competences:

Personal competences

(1) Learning and knowledge.
(2) Values.
(3) Communication and organizational skills.
(4) Knowledge of different areas of the science of medicines.
(5) Understanding of industrial pharmacy.

Patient care competences

(6) Patient consultation and assessment.
(7) Need for drug treatment.
(8) Drug interactions.
(9) Provision of drug product.
(10) Patient education.
(11) Provision of information and service.
(12) Monitoring of drug therapy.
(13) Evaluation of outcomes.

Most of those competencies are the same as described in Global Competency Framework, which was published by the FIP [6].

Respondents were asked to rank the proposals for competences with a Likert scale:

(1) Not important = Can be ignored.
(2) Quite important =Valuable but not obligatory.
(3) Very important = Obligatory with exceptions depending upon field of pharmacy practice.
(4) Essential = Obligatory.

The assessment methodology was based on that used by the MEDINE [36]; the even-numbered Likert scale was the same as that used by MEDINE. A pilot MEDINE experiment using a 5-point Likert scale, with a rank 3 = “neutral”, showed that respondents tended to “opt out” by replying with rank 3 throughout (M.T. Ross and A. Cummins, MEDINE, personal communication, 2012).

Respondents had the possibility to opt for “I cannot rank this competence” or to leave the answer blank. Finally, they could add their comments.

The distribution of surveymonkey to potential respondents was organized by the PHAR-QA regional directors, *viz* for northern Europe J. Hirvonen, for eastern B. Bozic, for western D. Rekkas, and for southern: A. Sanchez-Pozo. The stakeholders (EPSA, PGEU, EAHP, and EIPG) also distributed the questionnaire to their members. More than one-off emailing was required to obtain some balance in distribution of the profiles of the respondents; numerous telephone contacts and personal contacts were also made. The numbers of respondents snowballed through individual, local contacts.

Results are presented here in the form of scores based on the methodology used in MEDINE: score = (frequency rank 3 + frequency rank 4) as % total.

For example: data for community pharmacists ranking competence number 1:

**Table:** 

Rank	Frequency	
1	3	
2	121	
3	480	
4	622	
Total = 1226	*f* 3 + *f* 4 = 1102	Score = (1102/1226) × 100 = 90%

Scores give more granularity and a better pictorial representation; they represent “obligatory” rankings. A comparison with medians and means is given in the annex.

## 3. Statistical Analysis

Data presented in this paper are for:

- Overall rankings by six groups of respondents. These are given as means and scores. Although the parametric use of means was probably robust enough under the circumstances, means are given as an indication only and differences were determined using non-parametric methods (see below).
- Comparisons of ranking by community pharmacists with that of the 5 other professional groups of respondents

The differences between rankings of competences or between rankings by different categories of respondents were determined by the chi-square test (confidence level 95%).

Estimated sample size was calculated with a 95% confidence interval and a 10% error [37]. The confidence interval (also called margin of error) is the “plus-or-minus”. The confidence level is a measure of confidence. It is expressed as a percentage and represents how often the true percentage of the population who would pick an answer lies within the confidence interval. Most researchers use the 95% confidence level. For example: for community pharmacists (estimated population size: 400,000, 95% confidence interval and 10% confidence interval (margin of error)), the minimal sample size is 97. With a sample of 258 out of 400,000, a confidence level of 95% and a 10% error, for a score of 90% the confidence interval is 4, thus giving a score range of 86%–94%.

## 4. Results

There were 1613 entries in the *surveymonkey* questionnaire. Of these 1613, 1245 (77%) went beyond the profile description questions (first 6 questions on occupation, *etc.*) and ranked the competence ranking questions (competence clusters 7 through 19).

The numbers of the respondents in the 6 groups are given in Table 1. The relative size of the professional groups was: students > community pharmacists = academics > hospital pharmacists = industrial pharmacists > pharmacists working in other professions. The “other” group included pharmacists working in government agencies (regulatory affairs…), in wholesale, in marketing and sales, *etc.* In all groups sample sizes were well above calculated minimal sampling size (Table 1).

**Table 1:** Respondents by professional group, and sampling rates.

Professional Groups	Number of Respondents	%	Estimated EUROPEAN POPULATION (× 1000)	Calculated Minimal Sample Size (95% Confidence Level, 10% Error)
Community pharmacists	258	20.7	400 (PGEU)	97
Hospital pharmacists	152	12.2	12 (EAHP)	96
Industrial pharmacists	135	10.8	10 (EIPG)	96
Others	77	6.2	?	?
Breakdown of “others”				
Regulatory affairs, government	27	-	?	?
Consultancy	10	-	?	?
Wholesale, marketing, distribution	10	-	?	?
Lobbyist, NGO	6	-	?	?
Pharmacy chamber, society, association	5	-	?	?
Healthcare insurance agency	1	-	?	?
Not specified	18	-	-	-
Students	382	30.7	200 (PHARMINE)	96
Academics	241	19.4	10 (PHARMINE)	96
Total	1245	100	400 + 12 + 10 + 200 + 10 = 632	97

The ranking of the majority of the 1245 respondents (rank 3 + rank 4: 69.7%, Table 2) showed that the respondents considered the proposed competences were obligatory for pharmacy practice. 12% considered that competences were not important (rank 1), could not rank or left blanks. 9% either could not rank or left blanks.

**Figure 2:**
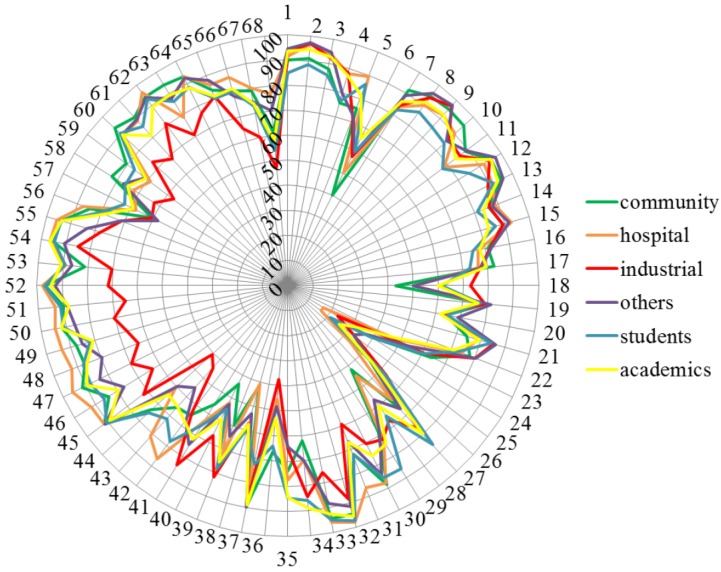
Ranking of the 68 competences by the 6 groups of respondents (community pharmacists: green, industrial pharmacists: red, hospital pharmacists: orange, others: purple, students; blue, academics: yellow). Numbers on the circumference refer to competences (1 through 68). Numbers on the vertical axis refer to % score (0 through 100).

Figure 2 shows the ranking of the 68 competences by the 6 groups of respondents. There was overall agreement between groups. Scores greater than 90% were observed for competences in groups 7, 8, 9, 10, 14, 15 and 17, and scores less than 50% for competences in groups 7, 9, 10, 11 and 12. These results indicate that some competences are not considered important although the group in general it is.

**Table 2:** Global ranking for entire population of respondents, *n* = 1245.

Rank	Number	%
1	2470	2.9
2	14,933	17.6
3	30,132	35.6
4	29,194	34.1
Cannot rank	1764	2.1
Blank	6167	7.3
Theoretical total	= 68 × 1245 = 84,660	100%

Comparisons between community pharmacists and other groups are given below.

**Figure 3:**
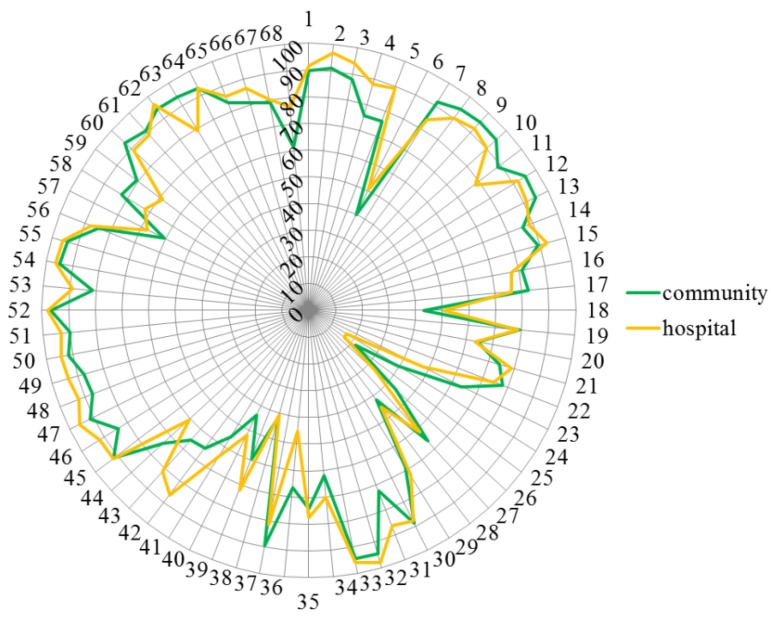
Comparisons of rankings by hospital (orange) and community pharmacists (green). Numbers on the circumference refer to competences (1 through 68). Numbers on the vertical axis refer to % scores (0 through 100).

Figure 3 shows that there was little difference in the rankings of hospital and community pharmacists. Ranking for competences 23, 24, 36 and 63 was community > hospital, and for competences 42, 43 and 68 community < hospital.

**Figure 4:**
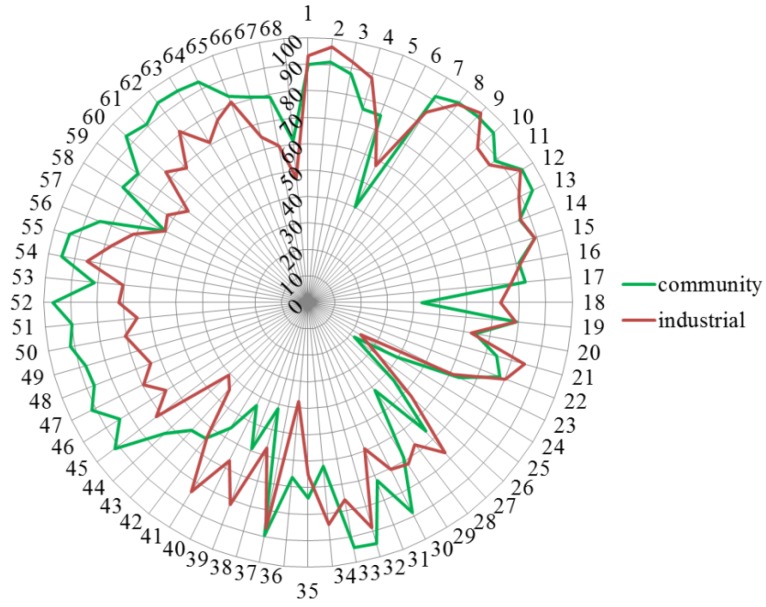
Comparisons of rankings by industrial (red) and community pharmacists (green). Numbers on the circumference refer to competences (1 through 68). Numbers on the vertical axis refer to % score (0 through 100).

Figure 4 shows that industrial pharmacists scored differently from community pharmacists. Ranking for competences 24, 30, 33, 36, 43–52, 55, 58, 60, 61, 63, 64, 66 and 67 was community > industrial, and for competences 6, 18, 28, 34 and 38–41 community < industrial.

**Figure 5:**
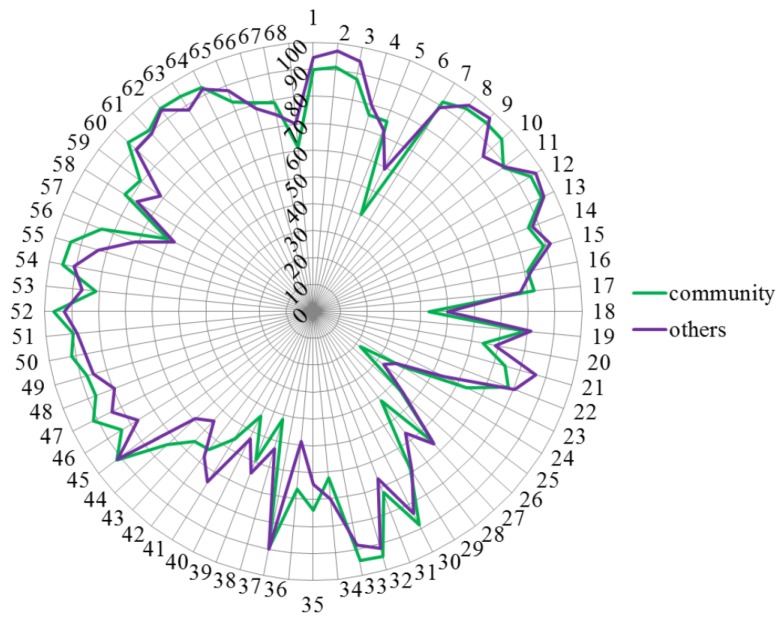
Comparisons of rankings by pharmacists working in other professions (purple) and community pharmacists (green). Numbers on the circumference refer to competences (1 through 68). Numbers on the vertical axis refer to % score (0 through 100).

Figure 5 shows that pharmacists working in professions other than community, industrial or hospital pharmacy gave scores similar to those of community pharmacists. Ranking for competence 36 was community > industrial, and for competences 6, 28 and 41 community < industrial.

**Figure 6:**
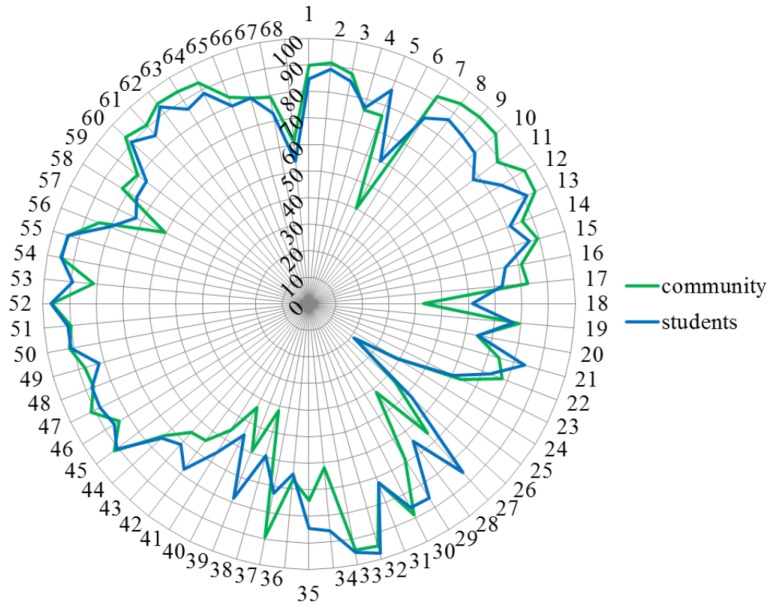
Comparisons of rankings by students (blue) and community pharmacists (green).

Figure 6 shows that students often gave higher scores than community pharmacists. Ranking for competence 37 was community > students, and for competences 6, 18, 27–29, 34, 38 and 39 community < students.

**Figure 7:**
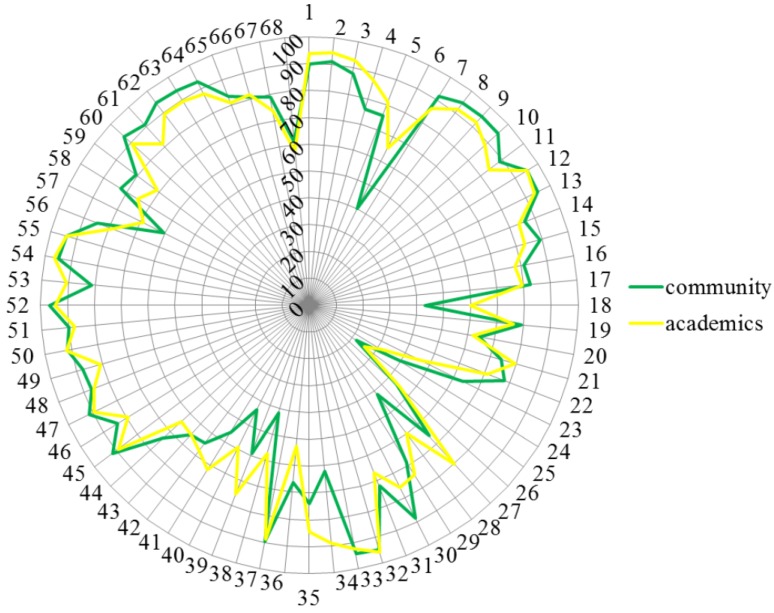
Comparisons of rankings by academics (yellow) and community pharmacists (green). Numbers on the circumference refer to competences (1 through 68). Numbers on the vertical axis refer to % score (0 through 100).

Academics often scored higher than community pharmacists. Figure 7 shows that ranking for competence 23 was community > academics, and for competences 6, 18, 28, 34 and 38–41 community < academics.

**Figure 8:**
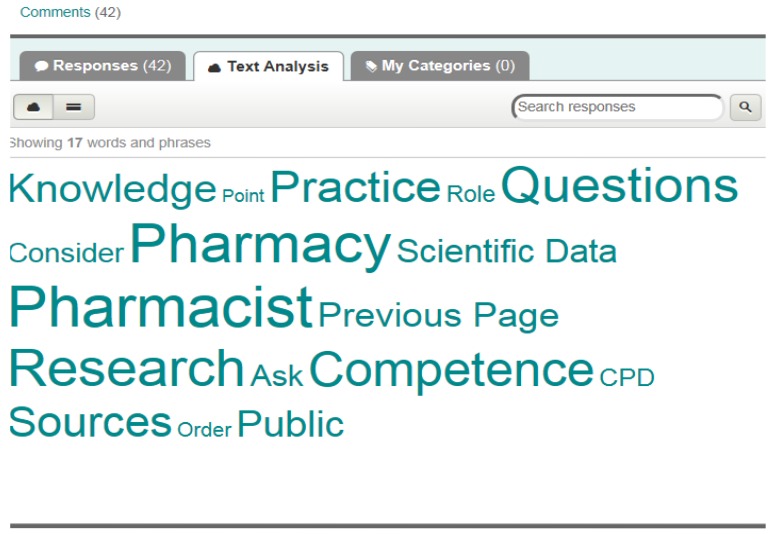
The *surveymonkey* text analysis tool (example for profile question group 10: Personal competences: learning and knowledge).

The *surveymonkey* text analysis tool allows the frequency of key words and key terms to be determined thus illustrating the relative importance of the terms. In Figure 8, the font size is proportional to number of citations.

Comments that occurred frequently included:

- Target audience
  ○ “…refer to daily work in a community pharmacy”
  ○ “focus on practicing pharmacists”
  ○ “for specialists”
  ○ “Not really the role of primary care, but important for some knowledge and awareness.”
  ○ “Things that every pharmacist should be familiar with and even more in patient care fields, as in hospital or community pharmacy.”
  ○ “For community pharmacists the above are essential, but for other pharmacists less.”
  ○ “Can imagine it to be important in hospitals...”
  ○ “For clinical and hospital pharmacists.”
- University level
  ○ “Competences recorded as ‘very important’ cannot be fully obtained on pre-graduate level and also postgraduate training is needed.”
  ○ “Competence 66 cannot be fully achieved during the pre-graduate training and requires also postgraduate education.”
- Difficulties in application
  ○ “Are subject areas professional competences?”
  ○ “If not commercially available I would contend that we should change what we are prescribing. I do not believe in ‘specials’ which in the UK are abused and contribute hugely and inappropriately to our drugs bill.”
  ○ “There are always people who need some special drug which is not commercially available.”
  ○ “Not sure how most pharmacists would be able to manufacture?”
  ○ “General information on diet or exercise is important but the specific recommendations for the patient should be made by the experts in those areas (e.g. dietician or physiotherapist).”
  ○ “Information should be basically provided by doctors, before pharmacists.”
  ○ “I am not sure that pharmacists know current clinical guidelines. If medicine is prescribed we give it to patient.”
- Suggestions for further inclusions, *etc*.
  ○ “Acquire other competencies for new services like vaccinations in the pharmacy, screening tests (colon cancer, heart disease, COPD, *etc.*) Public Health services in general, NCD (non-communicable diseases)”
  ○ “Services like vaccinations, screenings (colon cancer, kidney, COPD, Heart disease, *etc.*) and others should become essential in the curriculum in order to be able to perform the services in the future.”
  ○ “Pharmacist should also provide information about medical devices and other items available in the pharmacy.”
  ○ “The knowledge on drug therapies and reactions on failing therapies are core fields for pharmacists.”
  ○ “Radio-pharmacy”
- Technical difficulties with the survey
  ○ “In my browser section 6 appears blank”
  ○ “Never ask 2 things in the same question…”
  ○ “No possibility of open-ended questions…”
- Language difficulties
  ○ “Too complicated for my simple English…”
  ○ “I cannot rank this competence for I do not fully understand the meaning of the competence.”

## 5. Discussion

The results show that competences in the areas of “drug interactions”, “need for drug treatment” and “provision of information and service” were ranked highest whereas those in the areas of “ability to design and conduct research” and “development and production of medicines” were ranked lower. For the latter two competences one out of six categories—industrial pharmacists—ranked higher than the other 5 groups. The impact of the professional group status on the ranking will be dealt with in a future paper.

The six groups were formed following the end of data collection from respondents. There was no prior separation into sampling groups and no selection. Comments received during European-wide data collection (unpublished) suggested that snowballing was occurring with respondents being recruited by colleagues and friends. Furthermore, the identity of the respondents was unknown; only the computer IP numbers were collected; several respondents could use the same computer. Thus, the requirement for anonymity in Delphi studies was maintained in the second phase *i.e.*, the European-wide survey. This was not the case in the first phase *i.e.*, the elaboration of the survey by three Delphi rounds within the consortium.

Another question that scored low was that concerned with the subject area “physics”. This, however, is not a competence as such. They were included as they are part of the EU directive on the sectoral profession of pharmacy [2]. The question to be asked here was more accurately “adequate knowledge of the following areas (physics…) in the science of medicines is necessary to support pharmaceutical practice” but once again one is not dealing with competences for practice. Perhaps the best way to consider this is to take the teaching of certain subject areas as an essential, integral part of the acquirement of given competences for practice. This is the position taken by FIP (2012 reference) when they propose that the foundations of quality in pharmacy education are science (or knowledge), practice and ethics. The two aspects “knowledge” and “practice” are well separated and several papers have dealt with the question of how practice relates to knowledge (e.g., Waterfield) and whether pharmacy is a knowledge/science-based profession. The European answer to this question would be “yes” with the proviso that the way in which individual member state countries link practice to knowledge/science is their responsibility and not that of the European Commission.

This freedom of action is also reflected in the issue that organization and management competencies are not included in the framework, nor are time management, financial issues, responsibility for processes and decisions, new tools in the pharmacy profession, such as marketing, category management, procurement, and reimbursement for services. It is judged that such issues are more national than European. Albeit, a question is asked on the “ability to identify the need for new services” with the possibility to develop the answer in the comments box. Several comments were received on future developments in pharmacy practice.

The main difference of the PHAR-QA with the PHARMINE survey is that the former is shorter and more concise. It is intended that the PHAR-QA framework—compared to other national frameworks for example in the UK (CoDEG cited above)—be short and concise and represent a harmonized European version that can be adapted to the national situation in a given member state. The use of the second phase Delphi process ensured that the PHAR-QA framework is consensual and harmonized throughout European countries. This was done by using extensive, random, snowballing recruitment. As stated above, the recruitment was not entirely random as it was distributed by PHAR-QA regional directors and stakeholders—all pharmaceutical in nature—and was thus aimed at a specific population. The survey aimed at balance throughout European countries, professional and age groups. This was largely attained although some groups (e.g., students) and some countries (e.g., Germany) were over-represented in terms of the number of actual respondents compared to the number of potential respondents.

There was a relatively large number of respondents who did not go beyond the profile questions (23%). These were mainly students and this may be related to issues with the English language. The question can be asked as to whether the respondents were suitably armed to reply to the questionnaire. It is unfortunate that 23% of respondents did not go beyond the first six profile questions. However of the 1245 respondents × 68 questions = 84,660 potential replies there were “only” 2.1% “cannot rank” and 7.3% blanks.

The number of respondents (1245) far exceeded the sample size number of 100 respondents estimated for a total population of 632,000 potential respondents. As the numbers in all six categories are large this will allow inter- and intra-group comparisons. In this article, we presented comparisons between ranking by community pharmacists and the ranking by the other 5 professional groups. Many other comparisons are possible such as 1st year students *versus* 5th/6th year students, academics with students, different age groups, *etc*. These will be the subject of further publications. One particular comparison is of great interest: that concerning the ranking in different countries. Ever since the pioneering work of Bourlioux and the founder members of the EAFP [38] there has been a move to harmonization of pharmacy education throughout the EU driven partly by the publication of EU directives on the sectoral profession of pharmacy [39]. It will be interesting to know whether professionals in different member states have (or have not) similar views on the importance of the different competences for practice.

Regarding statistics, as the ordinal data of the Likert scale has only 4 units (1, 2, 3 or 4), the score was an attempt to introduce more granularity into the results than can be obtained with the use of medians. Scores measure the degree to which competences are considered “obligatory” (ranks 3 and 4). Although this adds granularity it does not convert the ordinal data into ratio data.

The comments from the respondents raised several issues on English phraseology and idiom and these have been corrected in the second version. Words that have a loose definition such as “familiarity” were also removed. Questions that asked 2 separate sub-questions such as “ability to perform and interpret medical laboratory tests” were simplified.

## 6. Conclusions

The results show that competences in the areas of “drug interactions”, “need for drug treatment” and “provision of information and service” were ranked highest whereas those in the areas of “ability to design and conduct research” and “development and production of medicines” were ranked lower.

This PHAR-QA framework does not, however, replace member state law or the EU directive on qualifications for the sectoral profession of pharmacy. The PHAR-QA framework simply represents the consensual opinion of several hundred European pharmacy professionals, academics and students.

## 7. Perspectives

The project started in October 2012 and will finish in March 2016, thus it is now entering its critical, final stage.

On the basis of the results above PHAR-QA has now produced a fifth version of the competence framework taking into account:

- The ranking of the fourth version of the framework presented in this paper
- The comments of the respondents, namely
  ○ Need for simplified construction of questions
  ○ Attention given to use of easy to understand English
- The question “did we miss anything?” with suggestions for competences to be included (open-ended question)

The revised version of the question is available and readers are invited to respond [40].

The final PHAR-QA framework will be exploited by EAFP that will propose its use in European pharmacy departments and suggest the modalities through which it could be introduced. In a later stage efforts will be made to introduce this competence framework approach to other aspects of education such as continuing professional development and to monitoring of practice.

## Appendix

**Table A1:** Ranking data for 68 competences (*n* = 1245 respondents).

	Number of Competence	Mean Ranking	Median Ranking	Score 3% + 4%
7. Personal competences: learning and knowledge.				
1. Ability to identify learning needs and to learn independently (including continuous professional development (CPD)).	1	3.4	4	89.89
2. Analysis: ability to apply logic to problem solving, evaluating pros and cons and following up on the solution found.	2	3.5	4	92.70
3. Synthesis: capacity to gather and critically appraise relevant knowledge and to summarize the key points.	3	3.4	4	89.70
4. Capacity to evaluate scientific data in line with current scientific and technological knowledge.	4	3.2	3	81.38
5. Ability to interpret preclinical and clinical evidence-based medical science and apply the knowledge to pharmaceutical practice.	5	3.2	3	81.02
6. Ability to design and conduct research using appropriate methodology.	6	2.7	3	55.47
7. Ability to maintain current knowledge of relevant legislation and codes of pharmacy practice.	7	3.3	3	85.96
8. Personal competences: values.				
1. Demonstrate a professional approach to tasks and human relations.	8	3.4	4	91.09
2. Demonstrate the ability to maintain confidentiality.	9	3.5	4	91.74
3. Take full personal responsibility for patient care and other aspects of one’s practice.	10	3.4	4	88.43
4. Inspire the confidence of others in one's actions and advice.	11	3.2	3	82.84
5. Demonstrate high ethical standards.	12	3.6	4	91.88
9. Personal competences: communication and organizational skills.				
1. Effective communication skills (both orally and written).	13	3.4	4	92.60
2. Effective use of information technology.	14	3.1	3	84.63
3. Ability to work effectively as part of a team.	15	3.3	3	87.76
4. Ability to identify and implement legal and professional requirements relating to employment (e.g., for pharmacy technicians) and to safety in the workplace.	16	3.1	3	78.43
5. Ability to contribute to the learning and training of staff.	17	3.0	3	77.46
6. Ability to design and manage the development processes in the production of medicines.	18	2.7	3	56.59
7. Ability to identify and manage risk and quality of service issues.	19	3.1	3	77.99
8. Ability to identify the need for new services.	20	2.8	3	64.00
9. Ability to communicate in English and/or locally relevant languages.	21	3.2	3	80.67
10. Ability to evaluate issues related to quality of service.	22	2.9	3	75.07
11. Ability to negotiate, understand a business environment and develop entrepreneurship.	23	2.7	3	56.62
10. Personal competences: knowledge of different areas of the science of medicines.				
1. Plant and animal biology.	24	2.2	2	32.87
2. Physics.	25	2.0	2	23.65
3. General and inorganic chemistry.	26	2.5	2	46.50
4. Organic and medicinal/pharmaceutical chemistry.	27	3.1	3	75.26
5. Analytical chemistry.	28	2.7	3	56.29
6. General and applied biochemistry (medicinal and clinical).	29	3.0	3	75.74
7. Anatomy and physiology; medical terminology.	30	3.2	3	82.86
8. Microbiology.	31	2.9	3	71.21
9. Pharmacology including pharmacokinetics.	32	3.7	4	95.21
10. Pharmacotherapy and pharmaco-epidemiology.	33	3.6	4	91.98
11. Pharmaceutical technology including analyses of medicinal products.	34	3.2	3	78.24
12. Toxicology.	35	3.1	3	77.92
13. Pharmacognosy.	36	2.7	3	56.07
14. Legislation and professional ethics.	37	3.3	3	83.13
11. Personal competences: understanding of industrial pharmacy.				
1. Current knowledge of design, synthesis, isolation, characterization and biological evaluation of active substances.	38	2.6	3	52.39
2. Current knowledge of good manufacturing practice (GMP) and of good laboratory practice (GLP).	39	3.0	3	72.60
3. Current knowledge of European directives on qualified persons (QPs).	40	2.6	3	54.44
4. Current knowledge of drug registration, licensing and marketing.	41	2.9	3	67.36
5. Current knowledge of good clinical practice (GCP).	42	3.0	3	71.96
12. Patient care competences: patient consultation and assessment.				
1. Ability to perform and interpret medical laboratory tests.	43	2.9	3	66.46
2. Ability to perform appropriate diagnostic or physiological tests to inform clinical decision making e.g., measurement of blood pressure.	44	2.8	3	66.27
3. Ability to recognize when referral to another member of the healthcare team is needed because a potential clinical problem is identified (pharmaceutical, medical, psychological or social).	45	3.4	4	88.86
13. Patient care competences: need for drug treatment.				
1. Retrieval and interpretation of relevant information on the patient's clinical background.	46	3.2	3	82.23
2. Retrieval and interpretation of an accurate and comprehensive drug history if and when required.	47	3.4	4	87.83
3. Identification of non-adherence and implementation of appropriate patient intervention.	48	3.3	3	84.80
4. Ability to advise to physicians and—in some cases—prescribe medication.	49	3.2	3	83.10
14. Patient care competences: drug interactions.				
1. Identification, understanding and prioritization of drug-drug interactions at a molecular level (e.g., use of codeine with paracetamol).	50	3.5	4	89.35
2. Identification, understanding, and prioritization of drug-patient interactions, including those that preclude or require the use of a specific drug (e.g., trastuzumab for treatment of breast cancer in women with HER2 overexpression).	51	3.4	4	87.51
3. Identification, understanding, and prioritization of drug-disease interactions (e.g., NSAIDs in heart failure).	52	3.6	4	93.61
15. Patient care competences: provision of drug product.				
1. Familiarity with the bio-pharmaceutical, pharmacodynamic and pharmacokinetic activity of a substance in the body.	53	3.3	3	85.62
2. Supply of appropriate medicines taking into account dose, correct formulation, concentration, administration route and timing.	54	3.6	4	94.03
3. Critical evaluation of the prescription to ensure that it is clinically appropriate and legal.	55	3.5	4	91.87
4. Familiarity with the supply chain of medicines and the ability to ensure timely flow of drug products to the patient.	56	3.1	3	80.26
5. Ability to manufacture medicinal products that are not commercially available.	57	2.9	3	66.57
16. Patient care competences: patient education.				
1. Promotion of public health in collaboration with other actors in the healthcare system.	58	3.1	3	75.53
2. Provision of appropriate lifestyle advice on smoking, obesity, *etc*.	59	3.0	3	73.07
3. Provision of appropriate advice on resistance to antibiotics and similar public health issues.	60	3.3	3	88.66
17. Patient care competences: provision of information and service.				
1. Ability to use effective consultations to identify the patient's need for information.	61	3.2	3	84.84
2. Provision of accurate and appropriate information on prescription medicines.	62	3.5	4	91.81
3. Provision of informed support for patients in selection and use of non-prescription medicines for minor ailments (e.g., cough remedies...).	63	3.4	4	86.09
18. Patient care competences: monitoring of drug therapy.				
1. Identification and prioritization of problems in the management of medicines in a timely manner and with sufficient efficacy to ensure patient safety.	64	3.3	3	89.01
2. Ability to monitor and report to all concerned in a timely manner, and in accordance with current regulatory guidelines on Good Pharmacovigilance Practices (GVPs), Adverse Drug Events and Reactions (ADEs and ADRs).	65	3.2	3	82.35
3. Undertaking of a critical evaluation of prescribed medicines to confirm that current clinical guidelines are appropriately applied.	66	3.1	3	79.88
19. Patient care competences: evaluation of outcomes.				
1. Assessment of outcomes on the monitoring of patient care and follow-up interventions.	67	3.0	3	74.14
2. Evaluation of cost effectiveness of treatment.	68	2.7	3	59.60
